# Deformation Behavior of β Phase in a WE54 Magnesium Alloy

**DOI:** 10.3390/ma16041513

**Published:** 2023-02-11

**Authors:** Bijin Zhou, Jie Wang, Hailong Jia, Ting Hao, Zhenwu Ma, Leyun Wang, Xiaoqin Zeng

**Affiliations:** 1 School of Mechanical Engineering, Suzhou University of Science and Technology, Suzhou 215009, China; 2 National Engineering Research Center of Light Alloy Net Forming, Shanghai Jiao Tong University, Shanghai 200240, China; 3 Key Laboratory of Automobile Materials of Ministry of Education, School of Materials Science and Engineering, Nanling Campus, Jilin University, Changchun 130025, China

**Keywords:** Mg–Y–Gd–Nd alloy, second phase, microstrain, synchrotron radiation

## Abstract

Second phases play a significant role in the development of high-performance magnesium alloys with rare earth elements. Here, in situ tensile tests combined with synchrotron radiation were carried out to investigate the deformation behavior of β phases in a WE (Mg–Y–Gd–Nd) alloy. By lattice strain analysis, it was found that micro load continuously transferred from the soft α-Mg matrix to the hard β phases during the whole plastic deformation, while this behavior was much more obvious at the beginning of deformation. Based on diffraction peak broadening, Williamson–Hall (W–H) plotting was used to study the microstrain of β phases. The results showed that the microstrain of β phases increased rapidly within 4% plastic strain and reached the maximum at plastic strain of ~6.5%. Since the β phases acted as hard phases, the microstrain was considered as a sign of the stress concentration near phase interfaces. It was also suggested that the effective release of local stress concentration at the β/α-Mg interface benefited the ductility of the WE alloy by the plastic deformation of β phases and phase interface sliding.

## 1. Introduction

The aging behaviors of magnesium alloys with rare earth elements (Mg–RE) have received considerable attention over the last decade because ultrahigh-strength Mg–RE alloys inevitably require precipitate regulation [1,2]. Up to now, the understanding of the precipitation sequences of main series of Mg alloys has been relatively comprehensive, where Mg–Y–Nd alloys in a supersaturated solid solution state (SSSS) are often subjected to phase transformation during isothermal heating: SSSS → ordered GP zones (zig-zag shape) → β″ (Mg_3_Nd, hcp structure) → β′ (Mg_12_YNd, orthorhombic structure)→ β_1_ (Mg_3_(Nd, Y), fcc structure) → β (Mg_14_Nd_2_Y, fcc structure) [3,4]. Moreover, the corresponding crystal structures of the various types of precipitates have been well characterized [5,6].

As metastable phases, β″, β′, and β_1_ are much tinier and denser than the equilibrium phase, β [7,8]. Nie and Muddle et al. [9] reported that β_1_ and β′ dominate the aging hardening of WE alloys. Meanwhile, it was revealed that both β_1_ and β′ have an orientation parallel to the {10.0} prismatic planes of Mg, a morphology that can effectively hinder basal dislocation gliding to enhance the strength of Mg materials. For the β phase, several recent works found that the Mg–RE alloys with the equilibrium phase can also achieve a combination of high strength, high toughness, and exceptional thermal stability [10,11,12,13], even though they are in an over-aged state due to precipitate coarsening. For example, Liu et al. [11] attributed the high strength and toughness properties of a Mg–11.8Gd–1.9Er–0.4Zr (wt.%) alloy (yield strength ~455 MPa and elongation ~12.0%) to ultrafine α-Mg grains and homogeneous β phases. Similarly, a Mg–8Gd–1Er–0.5Zr (wt.%) alloy containing intergranular β phases (100 nm–200 nm) and fine α-Mg grains also possessed impressive mechanical performance [12]. Although the importance of β phases in the optimization of strength and ductility for Mg alloys has been emphasized, the specific role of β phases during deformation has not been clarified so far.

In situ testing coupled with synchrotron diffraction and/or scanning electron microscopy (SEM) enables one to monitor the deformation behaviors of Mg alloys [14,15,16,17,18]. Lentz et al. [14] reported that the tension twinning activity in extruded WE54 alloys increased with the aging process because of the consumption of the solutes Y and Nd. Although the intragranular plate-shaped precipitates have a significant hardening effect on basal dislocation, the precipitates formed at grain boundaries (GBs) do not enhance dislocation slip in the WE54 alloy. Using in situ SEM, Sarvesha et al. [18] found that the Mg_17_Al_12_ phase in the AZ91 alloy fractures at the beginning of plastic deformation, suggesting that the Mg_17_Al_12_ phase does not undergo much load transfer from the α-Mg phase. In our previous work [19], in situ synchrotron X-ray diffraction was carried out to study the deformation behavior of Mg–Nd alloys with and without β_1_ phases. The lattice strain evolution indicated that the β_1_ phases did not present the typical load-transfer effect, as found in many other precipitate-hardened Mg alloys. Besides this, the deformation behavior of quasicrystal I-phase in an extruded Mg–6Zn–1Y (wt.%) alloy was systematically investigated by Garcés et al. [17] using synchrotron radiation and acoustic emission.

In the present work, the deformation behavior of β phase in an extruded WE alloy was studied using a combination of in situ tensile tests and synchrotron radiation. It was found that β phases generated during hot extrusion uniformly distribute over the fine-grained Mg matrix and that the prepared WE alloy has superior mechanical properties. Based on lattice strain and peak broadening analyses, the role of β phases in the high ductility of the alloy was elucidated.

## 2. Materials and Methods

A commercially available WE54 alloy with composition Mg–4.95Y–1.63Gd–1.91Nd–0.47Zr (wt.%) was used in this study (High Broad New Material Co. Ltd., Changsha, China). The as-received alloy was first solution treated at 525 °C for 8 h, then forward extruded to a bar of Φ10 mm with an extrusion ratio of 20:1 and extrusion speed of 1 mm/s. The temperature of the extrusion die was 500 °C. The extrusion microstructure was characterized by optical microscopy (OM, Zeiss Axio observer, Oberkochen, Germany), electron backscattered diffraction (EBSD, Oxford NordlysMax^2^, Oxford, UK), and transmission electron microscopy (TEM, JEOL-2100F, Akishima, Tokyo, Japan). The details of sample preparation for characterizing microstructures were reported previously [20,21].

**Figure 1:**
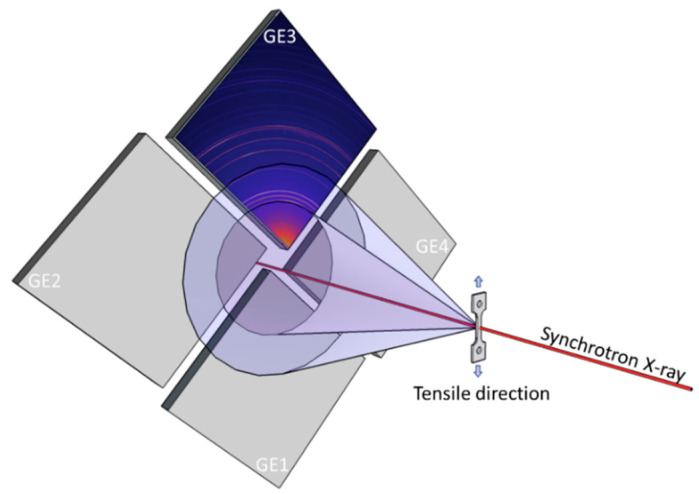
Schematic drawing of the in situ synchrotron radiation test.

Samples with nominal gauge dimensions of 5.0 mm (length) × 1.2 mm (width) × 1.0 mm (thickness) were cut from the extruded bar for the in situ tensile tests, with the tensile direction (TD) parallel to the extrusion direction (ED). Note that the samples were tested three times before in situ synchrotron radiation, and similar stress–strain curves were observed. The in situ synchrotron radiation experiments were carried out at the 1-ID beamline of the Advanced Photon Source at Argonne National Laboratory. A schematic drawing of the experimental setup is shown in Figure 1. During the in situ tensile tests ($\dot{\varepsilon }$ = 2 × 10^−4^ s^−1^), monochromatic X-rays with high beam energy of 71.68 keV (*λ* = 0.1730 Å) and beam size of 150 × 150 μm^2^ illuminated the gauge center of the samples and generated two-dimensional diffraction patterns in a row. Four amorphous Si area detectors (GE1~GE4) were used to collect the diffraction data. According to the calibration using standard CeO_2_ powder, the specimen-to-detector distance was measured as ~2.6 m. One-dimensional diffraction profiles (intensity vs. 2*θ*) were obtained by integrating the two-dimensional diffraction patterns over an azimuthal angle range of −10° to +10° around the tensile/axial direction.

During the tensile tests, diffraction peaks not only shift but also broaden. Each diffraction peak was fitted by the Gaussian function to gain the corresponding peak position (*θ*) and full width at half-maximum (*δ_t_*). The d-spacing of the {*hk.l*} diffraction plane (*d_hk.l_*) was calculated by Bragg’s law, *d_hk.l_* = *λ*/2sin*θ_hk.l_*. Then, the lattice strain of a reflection can be measured by *ε_hk.l_* = (*d_σ,hk.l_* − *d_0.hk.l_*)/*d_0,hk.l_* [22], where *d_0,hk.l_* and *d_σ,hk.l_* represent the calculated d-spacing of the {*hk.l*} plane before the tensile test and under load.

Diffraction peak broadening or the full width at half-maximum is usually ascribed to substructure size (*δ_D_*) and microstrain (*δ_ε_*). The combined effect was separated by standard W–H plotting [23], which can help quantify the microstrain evolution of the β phases. In this study, W–H plots show *δ_t_* vs. 4sin*θ_hk.l_* for different {*hk.l*} peaks. The slope of a W–H plot is proportional to the square root of the average dislocation density (*ρ*^0.5^), while the y-intercept is inversely proportional to the coherent scattering domain size [24].

## 3. Results and Discussion

**Figure 2:**
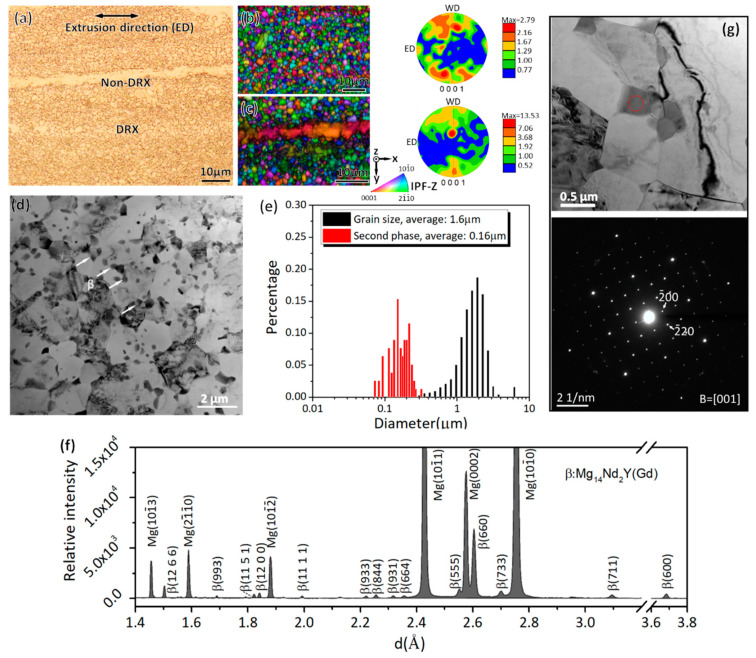
Microstructures of the hot-extruded WE54 alloy. (**a**) Optical micrograph; (**b**,**c**) IPF-Z maps coupled with image quality (IQ) showing the texture of the recrystallized grains and the elongated grains; (**d**) TEM image showing the distribution of the second phases in the matrix; (**e**) size distribution of the second phases and recrystallized α-Mg grains; (**f**) X-ray line profile where the diffraction peaks from the α-Mg and β-Mg_14_Nd_2_Y(Gd) were identified; (**g**) SAED results from the precipitate (see circled area) in the TEM bright field image.

The OM micrograph (Figure 2a) shows the extrusion microstructure of fine recrystallized grains and coarse elongated grains. Figure 2b presents the IPF-Z map of a region with recrystallized grains and the corresponding {0001} pole figure. The maximum texture intensity is only ~2.79 mrd, significantly lower than that of some extruded Mg alloys with rare earth elements [25,26,27]. However, the elongated grains, shown in Figure 2c, have a single orientation in which their *c*-axes are perpendicular to the extrusion direction of the sample. As a result, the Schmid factor (SF) of the basal dislocation slip in the elongated grains was near zero, which indicates that the strength of the alloy can be enhanced by texture strengthening [28].

The TEM image (Figure 2d) gives more details of the microstructure, in which a wealth of second phases decorates the matrix uniformly. The average diameter of the second phases is ~0.16 μm, which is one-tenth the average diameter of the recrystallized α-Mg grains, as shown in the statistics (Figure 2e). In the present study, the achievement of fine matrix grains has a direct relationship with the presence of the closely spaced second phases, which plays a role in effectively pinning GBs during dynamic recrystallization.

Figure 2f presents a synchrotron X-ray diffraction profile for phase identification. By comparing the standard powder diffraction file of Mg (#35-0821), six diffraction peaks of α-Mg were indexed. The remaining diffraction peaks can be ascribed to β-Mg_14_Nd_2_Y(Gd) based on its lattice parameter and crystal structure (*a* = 2.223 nm, FCC structure) [9,29]. This also shows that the intergranular second phases in Figure 2d were β-Mg_14_Nd_2_Y(Gd). Based on the selected area electron diffraction (SAED) results in Figure 2g, the lattice constant of the intergranular phases was calculated as 2.22 nm. This further determined the type of the second phases.

**Figure 3:**
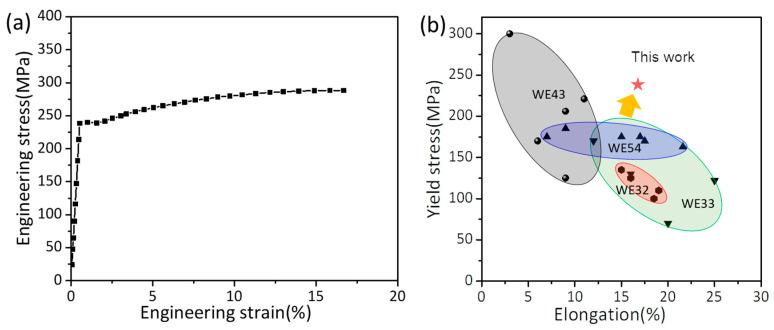
Mechanical properties of the extruded alloy at room temperature. (**a**) Engineering stress–strain curve; (**b**) comparison of the elongation and yield stress of different extruded WE alloys [14,31,32,33,34,35,36,37,38].

Figure 3a shows the engineering stress–strain curve from the in situ tensile test. The yield strength (YS), ultimate tensile strength (UTS), and elongation were ~240 MPa, ~288 MPa, and ~16%, respectively. The plastic strain from YS to UTS was measured as 15%, which indicates that the sample had a long uniform deformation stage. In addition, a plateau is observed near the yield region before strain hardening. Li et al. [30] reported that the yielding plateau at the initial stage of plastic deformation is mainly ascribed to the activation of, and rapid increase in, basal dislocations. Figure 3b presents the mechanical properties of different extruded WE alloys [14,31,32,33,34,35,36,37,38], suggesting the overall superior properties of the alloy in this work.

**Figure 4:**
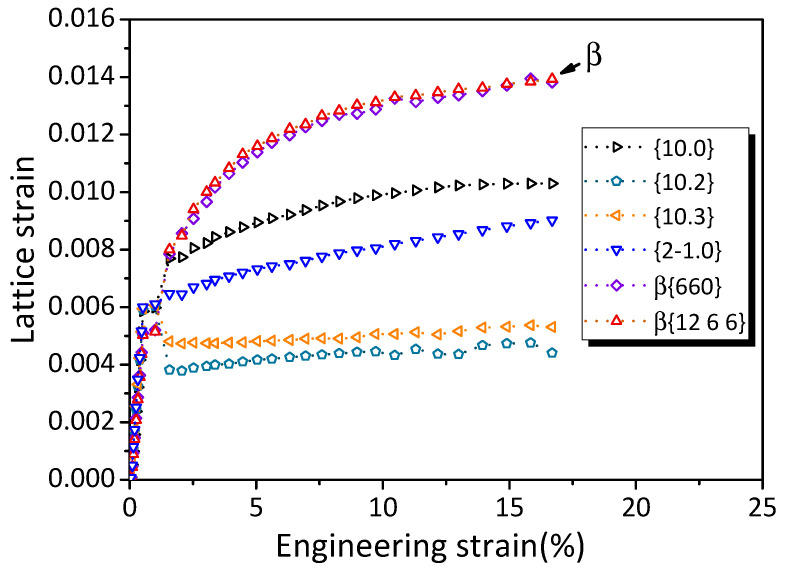
Lattice strain evolution with engineering strain for the β and α-Mg peaks along the tensile/axial direction.

Figure 4 shows the evolution of the lattice strain for the β phases and α-Mg with the engineering strain. Load-transfer behavior from the α-Mg matrix to the β phases was observed at the very beginning of the plastic deformation, which was reflected by the higher lattice strain of the {6 6 0}_β_ and {12 6 6}_β_ grain families than that of the α-Mg grain families. Conversely, our previous work [19] reported that β_1_ phases in a binary Mg–Nd alloy showed lattice strain close to that of α-Mg within 2% strain. This suggests that β phases likely have a more effective load-transfer effect than β_1_ phases.

**Figure 5:**
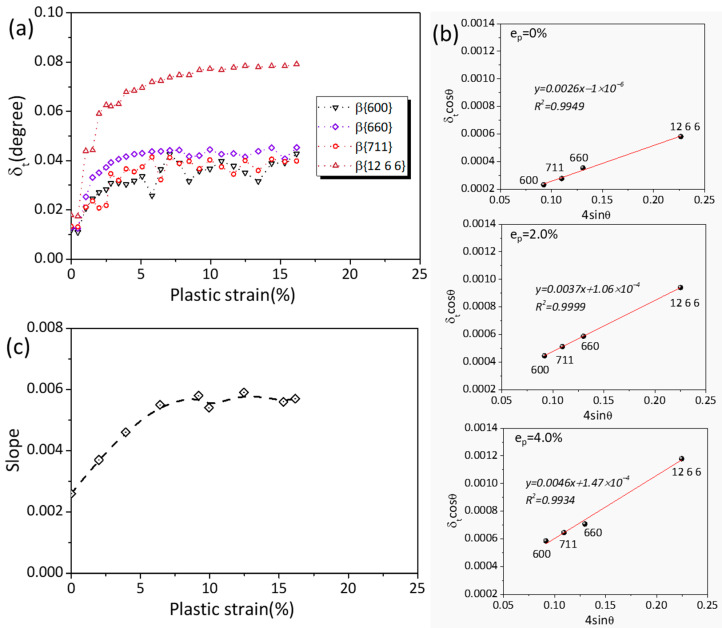
Diffraction peak analysis for the β phases. (**a**) Evolution of the measured FWHMs of the β peaks; (**b**) W–H plots for the β peaks at different plastic strains during the test. A strong variation in δ_t_cosθ as a function of 4sinθ can be seen. (**c**) W–H slope evolution of the β phases as a function of the plastic strain. The R-square values of the linear fitting for W–H analyses were above 0.95.

Figure 5a shows the δ_t_ values of four β peaks as a function of the plastic strain. The δ_t_ values of the β peaks also increased with the strain at early plastic deformation. Especially before 4% plastic strain, the δ_t_ of {12 6 6}_β_ increased from 0.018 to 0.059, which suggests that the microstrain of the β phases underwent extreme change during this regime.

To reveal the reason behind the unstable state of the β phases, W–H plots were made based on Figure 5a. The results at three strain levels of the studied alloy are shown in Figure 5b. It can be observed that the slope of the least-squares fitting lines, which reflects the microstrain of β phases [39], increased from 0.0026 to 0.0046 during the plastic deformation, while the intercept is very small. Because the β phases act as hard phases at early macro deformation (see Figure 4), the microstrain of the β phases should be mainly due to external factors rather than dislocation activation inside the β phases. It has been mentioned that the prepared alloy has a relatively random texture, so basal slip is facilitated and dominates the early deformation of the α-Mg matrix. Moreover, the fine matrix grains mean a reduced average free path of dislocations. Hence, the movement distance of basal slip was limited in the α-Mg grains and quickly hindered by phase interfaces or GBs. As a result of the rapid stress concentration at the β/α-Mg interface, the β phases showed a jump in microstrain.

**Figure 6:**
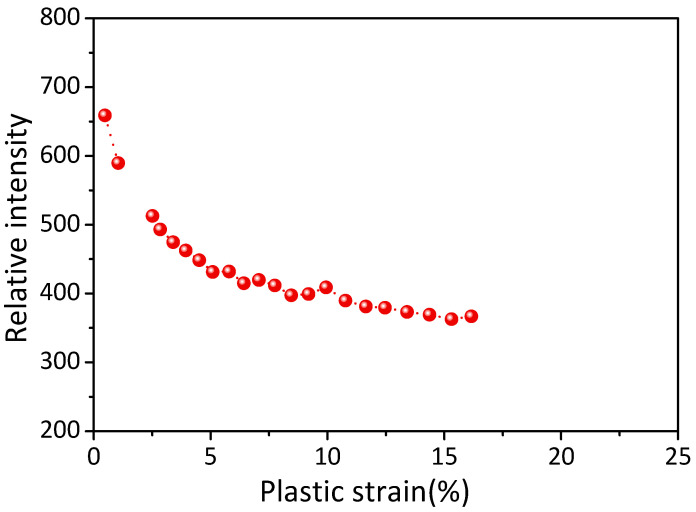
Intensity evolution of the {12 6 6}_β_ diffraction peak.

In addition, other slope values by peak broadening analyses are plotted as a function of strain in Figure 5c. As indicated, the maximum microstrain (reflected by the slope values) in the β phases is at ~6.5% plastic strain, implying that the effect of resistance against the dislocation movement by the β phases reaches its limitation and that the stress concentration near the β/α-Mg interface should be partly relieved somehow after ~6.5% plastic strain. Firstly, the phase interfaces of the alloy could slide in the fine microstructure. To support this point, the intensity evolution of the {12 6 6}_β_ peak was studied and is shown in Figure 6. As the peak intensity is proportional to the volume fraction of phases [40], the decreased peak intensity indicates the continuous volume reduction of β phases in the detected area. The volume reduction should be ascribed to the orientation change of the β phases, which implies a relative rotation between the β phase and matrix in this alloy. Local deformation was accommodated by phase interface sliding in the WE54 alloy at room temperature. Secondly, Guo et al. [41] reported that piled-up dislocations can be absorbed by GBs. This mechanism should also be involved and should relieve the localized stress concentration near the phase interfaces. In addition, the stress relaxation could be ascribed to the plastic deformation of the β phases at later deformation. In aged Mg–RE alloys, it has been experimentally confirmed that dislocation can shear across β′ [42,43] and β_1_ [19]. The deformability of the β phases was also verified by a recent first-principles study [44]. β phases should deform under high stress and subsequently provide an appropriate path to ease local stress concentration.

**Figure 7:**
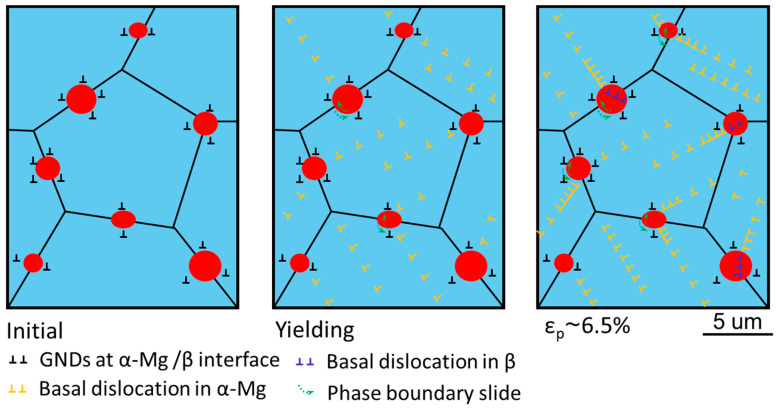
Schematic of the deformation mechanisms in the studied alloy.

To better understand the behavior of the β phases during tension, a schematic drawing related to the potential deformation mechanisms in the WE54 alloy is shown in Figure 7. It has been widely accepted that RE elements in solid solution states can significantly enhance non-basal dislocation activation [45]. During the hot extrusion in this study, the formation of β phases reduced the alloying element (Y, Gd, and Nd) concentration in the solid solution. Therefore, the promotion effect by RE elements on non-basal dislocation was inapparent in the prepared alloy, while basal dislocations were first activated and dominated in fine α-Mg grains upon yielding. Then, they piled up at the phase interfaces or GBs rapidly. Non-basal slip should play a role in accommodating the local deformation [20,46]. With the strain increases, the local stress concentration near phase interfaces and GBs was larger. The phase-interface-related movement and dislocation absorbing were simultaneously involved. At ~6.5% plastic strain, the plastic deformation of the β phases commenced. Besides the random texture of the recrystallized α-Mg grains, our study demonstrated that the good ductility of the material is also related to the effective stress relief by the β phases.

In this study, the β phases could not bring prominent strengthening effects because of the intergranular distribution and spherical shape [47]. However, it was speculated that further refinement of β phases could optimize the matrix microstructure of WE54 alloys, such as obtaining submicron α-Mg grains. As a result, the mechanical properties of the prepared alloy can be further improved. In addition, the strengthening mechanism will be different and interesting if the relative sizes of the β phase and Mg grains change. The effect of finer β phases with different distribution states on deformation behavior in Mg–RE alloys will be further studied, which could help to develop novel strategies for designing Mg alloys with high performance.

## 4. Conclusions

In this study, the role of β phases in the deformation of a hot-extruded WE54 magnesium alloy was studied using in situ synchrotron radiation. The main results are summarized as follows:

1. The prepared alloy with submicron intergranular β phases and fine α-Mg grains had superior mechanical properties (YS ~240 MPa and elongation ~16%).
2. The β phases acted as hard phases and showed an effective load-transfer effect.
3. The microstrain of the β phases quickly increased before 2% plastic strain, which can be ascribed to the rapid stress concentration at the β/α-Mg interface.
4. After ~6.5% plastic strain, the ability to hurdle the dislocation movement by β phases reached its limitation, and the stress concentration near the β/α-Mg interface was partly relieved, which helped develop the good ductility of the material.

## Data Availability

Data can be available on request.
